# Succinate dehydrogenase subunit B inhibits the AMPK-HIF-1α pathway in human ovarian cancer *in vitro*

**DOI:** 10.1186/s13048-014-0115-1

**Published:** 2014-12-10

**Authors:** Lilan Chen, Ting Liu, Shu Zhang, Jinhua Zhou, Yunfei Wang, Wen Di

**Affiliations:** Department of Obstetrics and Gynecology, Ren Ji Hospital, School of Medicine, Shanghai Jiao Tong University, Shanghai, 200127 P. R. China; Shanghai Key Laboratory of Gynecologic Oncology, Shanghai, 200127 P. R. China

**Keywords:** Succinate dehydrogenase subunit B, HIF-1α, AMPK, Gene silence, Gene overexpression, Ovarian carcinoma

## Abstract

**Background:**

Ovarian carcinoma is one of the most common gynecological cancers with high mortality rates. Numerous evidences demonstrate that cancer cells undergo metabolic abnormality during tumorigenesis in tumor microenvironment and further facilitate tumor progression. Succinate dehydrogenase (SDH or Complex II) is one of the important enzymes in the tricarboxylic acid (TCA) cycle. Succinate dehydrogenase subunit B (SDHB) gene, which encodes one of the four subunits of SDH, has been recognized as a tumor suppressor. However the role of SDHB in ovarian cancer is still unclear.

**Methods:**

Using the *SDHB* specific siRNA and overexpression plasmid, the expression of SDHB was silenced and conversely induced in ovarian cancer cell lines SKOV3 and A2780, respectively. The possible role of SDHB in ovarian cancer was investigated *in vitro*, using proliferation, migration and invasion assays. To explore the mechanism, proliferation and migration related proteins such as Bcl-2, cleaved caspase 3, p-ERK, MMP-2, and p-FAK were examined by western blot. P-P38, p-AMPKα, and HIF-1α were also examined by western blot. CoCl2 was used to induce HIF-1α expression in SKOV3 and A2780 cells.

**Results:**

*SDHB* silencing promoted cell proliferation, invasion, and migration, but inhibited apoptosis of SKOV3 and A2780 cells. In contrast, overexpression of SDHB inhibited cell proliferation, invasion, migration, and promoted apoptosis in SKOV3 cells. It was observed that up-regulation of Bcl-2 and MMP-2, activation of p-P38, p-ERK, and p-FAK, inhibition of cleaved caspase 3 in *SDHB*-silenced cells. Meanwhile, decreased Bcl-2 and MMP-2, inhibition of p-P38, p-ERK, and p-FAK, activation of cleaved caspase 3 were shown in *SDHB*-overexpressed SKOV3 cells. HIF-1α, an essential factor in tumor progression, was up-regulated in *SDHB*-silenced cells with the activation of p-AMPKα and down-regulated in *SDHB*-overexpressed cancer cells with the decreased p-AMPKα. And SDHB was proved to be decreased due to upregulation of HIF-1α expression in CoCl2-treated cancer cells.

**Conclusions:**

Our results firstly revealed that SDHB played a key role in cell proliferation, invasion, migration, and apoptosis of human ovarian carcinoma *via* AMPK-HIF-1α pathway. *SDHB*-overexpression might be a new approach to inhibit tumor progression in human ovarian carcinoma.

**Electronic supplementary material:**

The online version of this article (doi:10.1186/s13048-014-0115-1) contains supplementary material, which is available to authorized users.

## Background

Ovarian carcinoma is one of the leading causes of death among gynecological malignancies [1] that the most of ovarian carcinoma are firstly detected in advanced stages [2] and overall 5-year survival rate is as low as 40% [3]. Current therapeutic strategies against advanced stage ovarian carcinoma include surgical resection along with platinum-based chemotherapy. However, there are serious side effects from platinum-based drugs and surgical intervention [2]. Therefore, it is critical to understand the molecular events leading to initiation and progression of this devastating disease [4].

Succinate dehydrogenase (SDH), known as succinate-ubiquinone oxidoreductase, is a mitochondrial enzyme complex that catalyses the oxidation of succinate to fumarate in the citric acid cycle and participates in the electron transport chain [5]. Mutations of the *SDH* gene determine the genetic basis of paragangliomas/pheochromocytomas (PCCs/PGLs) [6,7] and associated tumour syndromes (Carney-Stratakis syndrome and Carney triad) [8]. Furthermore, mRNA expression of *SDHB* is decreased in recovering Ramos cells in childhood non-Hodgkin lymphoma (NHL) [9]. Reduced SDHB expression is associated with growth and de-differentiation of colorectal cancer cells [10]. In addition, loss of *SDHB* expression is associated with an adverse outcome in PCCs/PGLs, indicating SDHB might be a predictive marker of adverse outcome both in sporadic and familial PCC/PGL [11]. However, the role of *SDHB* in ovarian carcinoma tumourigenesis, especially its association with cellular proliferation, invasion, and migration are not fully elucidated.

Genetic analysis of hereditary paraganglioma reveals an activation of the hypoxia-response pathway [12]. Immunohistochemical analysis shows strong staining of hypoxia-inducible factor-1α (HIF-1α) and the angiogenic factor, vascular endothelial growth factor in a malignant sporadic pheochromocytoma caused by a germline missense mutation in the *SDHB* gene [13], suggesting that activation of the hypoxia-response pathway may be a common theme underlying SDH (and also FH) mutation [14,15].

Hypoxia-inducible factor-1 (HIF-1), consists of a constitutively expressed β-subunit and an inducib-expressed α-subunit [16], is a well-established mediator in hypoxia-response pathway. HIF-1α is accumulated under hypoxic conditions, which activates transcription of target genes involved in angiogenesis, energy metabolism, adaptive survival or apoptosis [17,18]. HIF-1α is highly expressed in ovarian cancer and is associated with tumour proliferation [19], invasion and metastasis [20,21]. AMP-activated protein kinase (AMPK) is a metabolic sensor that helps maintain cellular energy homeostasis and modulate metabolic stresses such as hypoxia and respiratory impairment. AMPK has been linked to the regulation of tumorigenesis [22] and HIF-1α mediates the growth advantage of tumours with reduced AMPK signaling [23]. However, the precise linkage between metabolism dysfunction (HIF-1α, AMPK) and the propensity for tumourigenesis has not been fully elucidated in ovarian carcinoma. In current study, we aimed to gain a better understanding of the consequences of *SDHB* alteration by gene silencing or overexpression in human ovarian cancer cell lines. Here, we provided the first time that *SDHB* silencing resulted in increased tumour cell proliferation, invasion, migration and decreased apoptosis. Conversely, overexpression of *SDHB* inhibited cell proliferation, invasion, migration, and promoted apoptosis. Further, HIF-1α and p-AMPKα were found to be upregulated in *SDHB-*silenced, but downregulated in *SDHB*-overexpressed cancer cells. Moreover, SDHB was downregulated by hypoxia mimetic CoCl2 in human ovarian cancer cells. Our data suggested that SDHB plays an essential role in ovarian cancer cell proliferation, invasion, migration and apoptosis *via* AMPK-HIF-1α pathway. *SDHB*-overexpression might be a potential therapeutic strategy to inhibit tumour progression in human ovarian carcinoma.

## Methods

### Tissue specimens and ethics

A total of 25 tissue specimens were collected from surgical patients enrolled in this study, including 7 normal human ovarian epithelium tissues and 18 ovarian carcinoma tissues between July, 2011 to August, 2012. In addition, 7 metastatic ovarian carcinoma were collected. All specimens, obtained during surgery (Department of Obstetrics and Gynaecology, Ren Ji Hospital, School of Medicine, Shanghai Jiao Tong University, China), frozen immediately in liquid nitrogen and stored at −80°C until analysis. All carcinoma patients were newly diagnosed with ovarian tumours, and received no chemical therapy prior to surgery. The study was approved by the Institutional Review Board of Ren Ji Hospital, Shanghai Jiao Tong University School of Medicine. Written informed consents were obtained from all patients. Clinical investigation was conducted according to the principles expressed in the Declaration of Helsinki.

### Cell lines and cell culture

Human epithelial ovarian adenocarcinoma cancer (EOC) cell lines SKOV3 and A2780 obtained from the Cell Bank Chinese Academy of Science (Shanghai, China), cultured in RPMI1640 medium (Hyclone) supplemented with 10% foetal bovine serum (Gibco). These cells were incubated at 37°C in a humidified atmosphere of 95% air and 5% CO_2_. The medium was replaced every 24 h. Hypoxic cells were incubated in the same conditions but in a hypoxic atmosphere with different concentrations of cobalt chloride (CoCl2, Sigma) for 24 h.

### Transient transfection of SKOV3 and A2780 cells with *SDHB* siRNA oligonucleotides

Transient small interfering RNA (siRNA) oligonucleotides were synthesized from Shanghai Integrated Biotech Solutions Co.Ltd. The target sequences were as follows: siSucA *5'-GAT TAA GAA TGA AGT TGA CTC-3'* [24]*,* siSucC *5'-GCT CAG AGC TGA ACA TAA TT-3'* [24]. As a control for silencing, we constructed a negative control (NC) siRNA (*5’-UUC UCC GAA CGU GUC ACG UTT-3*’) that did not affect *SDHB* expression. Transient transfection was carried out using Lipofectamine 2000 reagent (Invitrogen, Carlsbad, CA) according to the manufacturer’s instructions. Cells were collected after 24 h for RNA extraction and 48 h for protein electrophoresis.

### Transient transfection of ovarian cancer cell SKOV3 with *SDHB* overexpression pIRES2-EGFP plasmid

The full length SDHB opening reading frame was sub-cloned into *pIRES2-EGFP* plasmid. The plasmid was purchased from Shanghai Integrated Biotech Solutions Co., Ltd. SKOV3 cells were transfected with the *pIRES2-EGFP SDHB* vector encoding *SDHB* cDNA using lipofectamine 2000 (Invitrogen) according to the manufacturer’s instructions. Vector-only transfecting cells were used as controls.

### RNA extraction and quantitative real-time reverse transcriptase PCR

Total RNA, extracted from cancer cell lines and tissue samples using TRIzol Reagent (Invitrogen, Carlsbad, CA), was reverse transcribed using a PrimeScript RT reagent Kit (TaKaRa, Shanghai, China). Resultant complementary DNAs (cDNAs) underwent quantitative real-time reverse transcriptase PCR using a SYBR Green PCR Master Mix Reagent Kit (TaKaRa). Real-time PCR and data collection were performed on an ABI 7500 real-time system (Applied Biosystems). The primers for *SDHB* were *5'- AAA TGT GGC CCC ATG GTA TTG-3'* (forward) and *5'-AGA GCC ACA GAT GCC TTC TCT G-3'* (reverse). The primers for *β-actin*, serving as the endogenous controls, were *5'-TGA CGT GGA CAT CCG CAA AG-3'* (forward) and *5'-CTG GAA GGT GGA CAG CGA GG-3'* (reverse). SDS v.1.4 (Applied Biosystems) software was used to perform comparative delta cycle threshold (Ct) analysis. To minimize random variation in cell experiments, each real-time PCR experiment was performed in triplicate.

### Succinate dehydrogenase activity assay

Succinate dehydrogenase activity was determined, using succinate dehydrogenase kit (Beijing Solarbio Science & Technology Co., Ltd, China). Protein level was quantified by BCA kit (Cwbiotech, China).

### ATP assay

Intracellular ATP concentrations were determined using phosphomolybdic acid colorimetric method (Nanjing Jiancheng Bioengineering Institute, China) according to the manufacturer’s protocols. Protein level was quantified by BCA kit (Cwbiotech, China).

### Cell proliferation analysis

Cell proliferation was assessed by Cell Counting kit-8 (CCK-8) (Dojindo). Briefly, 1 × 10^3^ SKOV3 and/ or A2780 cells were seeded into 96-well plates treated with *SDHB* siRNA oligonucleotides or *pIRES2-EGFP SDHB* vector. After 1, 2, 3, 4, 5 or 6 day treatment, 10 μl CCK8 mixed with 90 μl RPMI1640 was added to each well for further 1.5 h. The absorbance (450 nm) from each well was measured at the indicated time points.

### Cell invasion and migration assay

SKOV3 and A2780 cells (6 × 10^4^) (48 h post-transfection) were placed into the upper compartment of the chambers (8 μm pore size, Millipore) coated with 50 μl BD Matrigel (diluted 1:7 in serum-free medium) and placed into 24-well plates. Medium containing 10% foetal bovine serum was added to the lower chamber. After incubation at 37°C for 24 h, cells on the upper side of the membrane were removed using sterile cotton swabs. Cells adhering to the lower surface were fixed in 4% paraformaldehyde and stained with 0.1% crystal violet. Cells were counted using microscope (Nikon TE300, Tokyo, Japan) at 200× magnification. Five random fields were selected for examination on each membrane, and results were expressed in terms of the invasion cells per field. Each experiment was conducted in triplicate. Cell migration was performed using a similar approach without Matrigel coating, and the treated cells were incubated for 12 h.

### Western blot

Cells and tissue samples were lysed in RIPA buffer with PMSF as protease inhibitor (Beyotime, Shanghai, China). Proteins were separated by sodium dodecyl sulfate-polyacrylamide gel electrophoresis and transferred on NC or PVDF membrane (Millipore, Bedford, MA). After blocking with 5% dry milk in TBST for 2 h at room temperature, the membranes were incubated with the primary antibodies against SDHB (1:1000, Epitomics), β-tubulin (1:4000, Epitomics), β-actin (1:500, Abmart), caspase 3 (1:1000, Cell Signalling Technology), Bcl-2 (1:4000, Epitomics), MMP-2 (1:500, Abcam), FAK (1:1000, CST), p-FAK (1:1000, CST), AMPKα (1:1000,CST), p-AMPKα (1:1000, CST), GAPDH (1:4000, Abmart), P38 (1:1000, CST), p-P38 (1:1000, CST), ERK (1:1000, CST), p-ERK (1:1000, CST), HIF-1α (1:1000, Epitomics) in dilution buffer overnight at 4°C. Membranes were washed for three times with TBST, then were incubated with IRDye 800CW conjugated goat (polyclonal) anti-Rabbit IgG or anti-Mouse IgG (1:10000) antibodies for 1 h at room temperature. The expression of specific proteins was detected through the use of Odyssey system following the manufacturer's instructions.

### Statistical analysis

All data were calculated as the means ± standard error (SEM). An independent Student *t* test was used to compare the continuous variables between groups using GraphPad Prism 5.0 software (San Diego, CA). *P* < 0.05 was considered statistical significant.

## Results

### The effect of *SDHB* silencing on ATP and AMPK/P38 MAPK in human ovarian cancer cells

**Figure 1:**
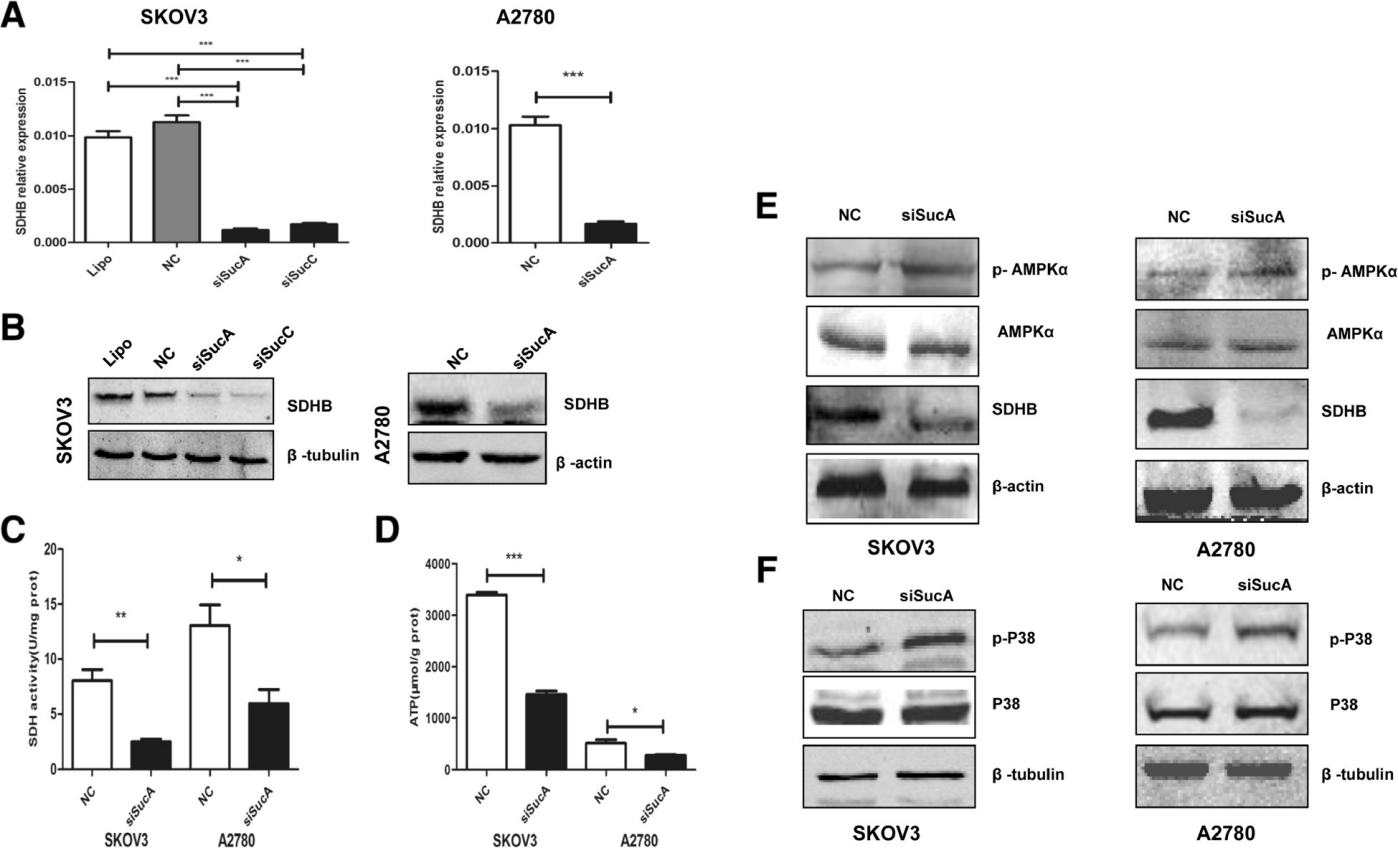
***SDHB*** **silencing in SKOV3 and A2780 cells.** SKOV3 and A2780 cells were treated with siSucA or siSucC oligonucleotides, **(A)** real-time PCR (24 h) and **(B)** western blot (48 h) were performed to detect *SDHB* mRNA and protein levels, respectively. 48 h after siRNA transfection, SDH activity **(C)** and ATP level **(D)** were examined, p-AMPKα, AMPKα **(E)** and p-P38, P38 **(F)** were measured in SKOV3 and A2780 cells. Mean ± SEM. *P < 0.05, **P < 0.01 and ***P < 0.001.

The role of SDHB on ATP and AMPK/P38 MAPK was examined using gene silencing strategy. The efficiency of *SDHB* silencing was confirmed by 24 h *SDHB* siRNA oligonucleotides treatment in SKOV3 or A2780 cells, mRNA level was reduced by 89.80% (siSucA *vs* NC in SKOV3), 84.89% (siSucC *vs* NC in SKOV3) (*P* < 0.001), and 83.88% (siSucA *vs* NC in A2780) (*P* < 0.001), respectively (Figure 1A). Furthermore, SDHB protein level and activity were also decreased following 48 h transfection (Figure 1B,C). Meanwhile, decreased ATP was observed in *SDHB*-silenced cells (Figure 1D). AMPK acts as an energy sensor that modulate metabolic stresses such as hypoxia and respiratory impairment [25,26], exogenous stimuli that increase AMP or decrease ATP could activate AMPK [27]. To examine the effect of *SDHB* silencing on AMPK activation, the level of phosphorylated AMPKα (p-AMPKα) was analysed. P-AMPKα was increased in the *SDHB*-silenced cells compared to NC group (Figure 1E). In addition, the AMPK/P38 MAPK signalling cascade stimulates glucose uptake during metabolic stress [28,29]. Moreover to confirm the effect of decreased AMPK activity in *SDHB*-silenced cells, phosphorylated p38 MAPK (p-P38 MAPK) level was also examined. P-P38 MAPK was increased in the *SDHB*-silenced cells compared to control cells (Figure 1F).

### *SDHB* silencing promoted ovarian cancer cell proliferation

**Figure 2:**
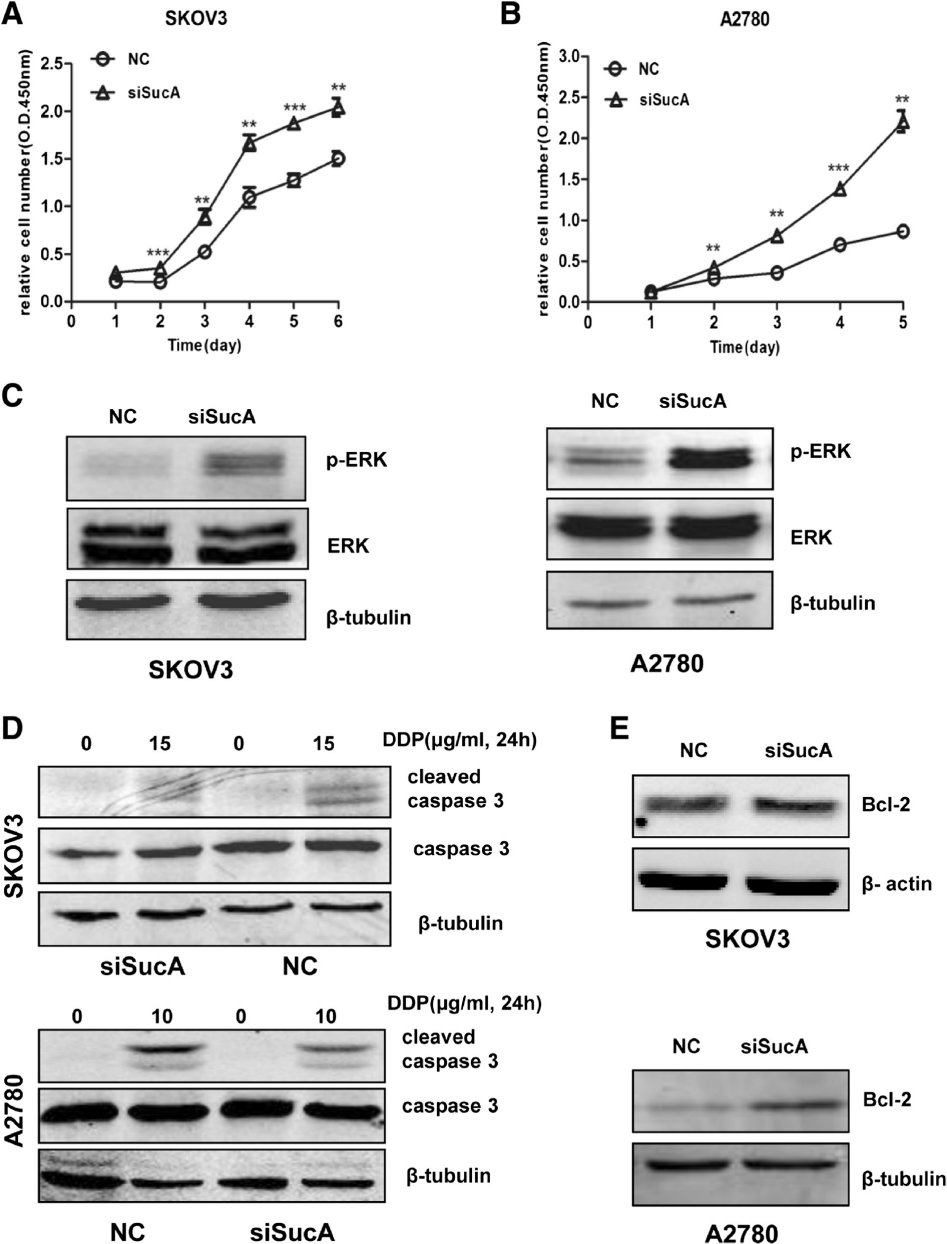
***SDHB*** **silencing contributed to ovarian cancer cell proliferation in SKOV3 and A2780 cells. (A, B)** Cell proliferation was assessed following *SDHB* silencing treatment for 6 days by CCK8. Mean ± SEM. *P < 0.05, **P < 0.01 and ***P < 0.001. **(C)** 48 h post transfection, p-ERK, ERK were examined by western blot. **(D, E)** 48 h post-transfection, SKOV3 and A2780 cells were treated with DDP at indicated concentration for 24 h, cleaved caspase 3, total caspase 3, and Bcl-2 were then analysed by western blot.

CCK8 was used to examine the influence of SDHB on cell proliferation. *SDHB* silencing significantly promoted ovarian cancer cell proliferation (*P* < 0.05) (Figure 2A, B). Extracellular signal-regulated kinase (ERK)1/2 mitogen-activated protein (MAP) kinase pathway plays a central role in cell proliferation control [30]. Multiple lines of evidence have implicated the ERK1/2 MAP kinase pathway in the control of cell proliferation [30,31]. ERK1 and ERK2 are activated in response to virtually all mitogenic factors [30]. ERK signalling is often up regulated in a diverse range of human cancers [32]. It was discovered that the level of p-ERK was increased in the *SDHB*-silenced cells compared to control (Figure 2C), indicating that inhibition of SDHB expression promoted tumour cell growth through ERK pathway in SKOV3 and A2780 cells.

### *SDHB* silencing prevented apoptosis in human ovarian cancer cells

Cleaved caspase-3, and Bcl-2 are representative proteins related to cell apoptosis. Cis-platinum (DDP, Sigma) is a chemotherapy for ovarian cancer, by forming a platinum complex inside the cell which binds to DNA and cross-links DNA and causes the cells to undergo apoptosis, or systematic cell death. Cleaved caspase-3 was detected in DDP treated cells for 24 h and its expression was decreased in *SDHB*-silenced cells (Figure 2D). Meanwhile, Bcl-2 was increased in *SDHB*-silenced cells (Figure 2E). The results suggested *SDHB* silencing could prevent ovarian cancer cell apoptosis.

### *SDHB* silencing promoted cell invasion and migration of SKOV3 and A2780 cells

**Figure 3:**
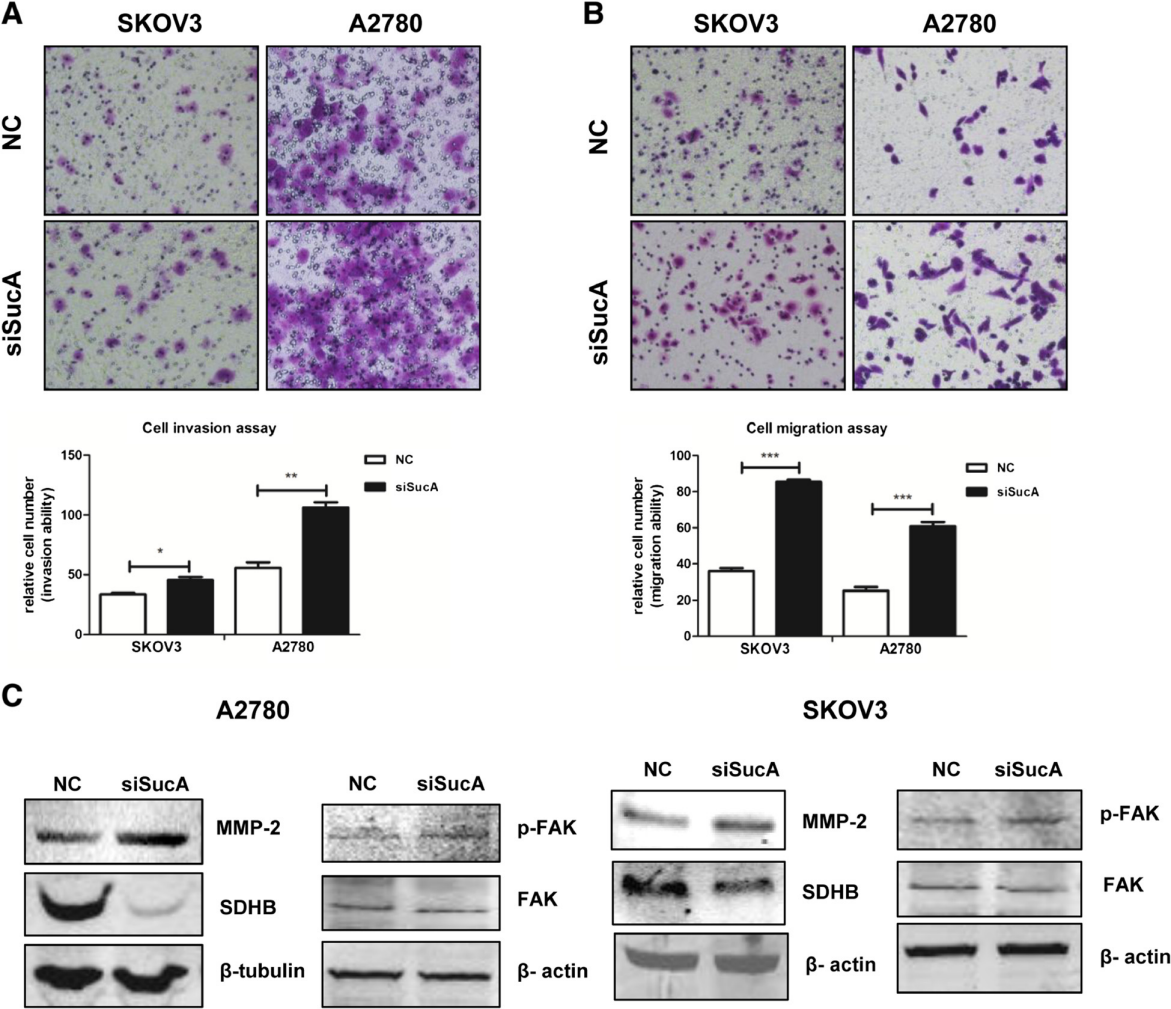
***SDHB*** **silencing promoted ovarian cancer cell invasion and migration.** The transwell assay and the Boyden Chamber test were used to evaluate cell invasion and migration after SKOV3 and A2780 cells were treated with *SDHB* siRNA for 48 h. The number of invaded and migrated cells was counted using a bright-field microscope (200×). Representative images and the relative cell invasion and migration rate were shown in **(A)** and **(B)**. Mean ± SEM. *P < 0.05, **P < 0.01, and ***P < 0.001. **(C)** 48 h after transfection, MMP-2 and p-FAK in *SDHB*-silenced A2780 and SKOV3 cells were analysed by western blot.

Invasion is among the most significant biological characteristics of malignant tumours. Matrigel cellular invasion assay showed that *SDHB* silencing promoted SKOV3 (*P* < 0.05) and A2780 (*P* < 0.01) invasiveness (Figure 3A). Migration assay showed that *SDHB* silencing upregulated migration ability of SKOV3 (*P* < 0.001) and A2780 cells (*P* < 0.001) (Figure 3B). The active, phosphorylated form of focal adhesion kinase (p-FAK) is a non-receptor protein tyrosine kinase that is involved in cell migration, proliferation, survival and metastasis [33,34]. The extent of p-FAK overexpression correlates with increased metastasis and decreased survival in human ovarian cancer [33,34]. MMP-2 degrades components of the extracellular matrix (ECM) proteins, contributing to tumour invasion and metastasis [35,36]. Up-regulation of p-FAK and MMP-2 expression was observed in *SDHB*-silenced cells (Figure 3C).

### *SDHB* overexpression inhibited cell proliferation and promoted apoptosis in SKOV3 cells

**Figure 4:**
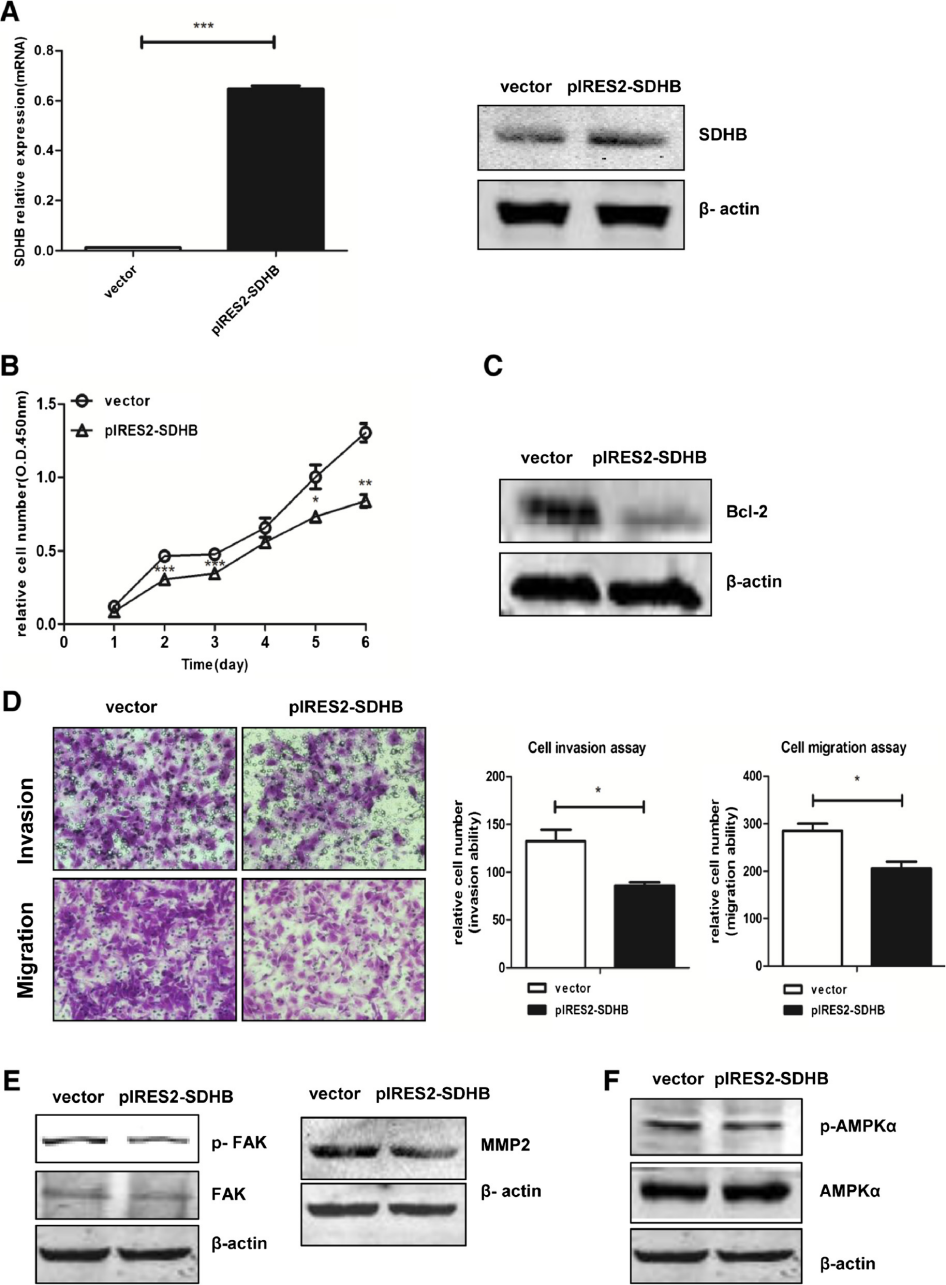
***SDHB*** **overexpression inhibited cell proliferation in SKOV3 cells. (A)** SKOV3 cells were treated with *pIRES2-SDHB* or empty vector, then *SDHB* mRNA (24 h after treatment) and protein (48 h after treatment) were analysed by real time-PCR or western blot, respetively. **(B)** 48 h after transfection, cell viability of *SDHB*-overexpressed SKOV3 cells was examined using CCK8 assay for another 6 days. **(C)** Bcl-2 was downregulated after 48 h transfection in SKOV3 with β-actin as a loading control by western blot. **(D)** SKOV3 cells were treated with *SDHB* overexpression plasmid for 48 h, cell invasion and migration ability was evaluated as described previously. **(E)** MMP-2 and p-FAK, FAK were also analysed by western blot. **(F)** p-AMPKα, AMPKα were measured. Mean ± SEM. *P < 0.05, **P < 0.01, and ***P < 0.001.

SKOV3 cell line was chosen to overexpress SDHB since SDHB expression in SKOV3 was lower than that in A2780 (see Additional file 1: Figure S1). After treated with *SDHB*-overexpressed plasmid for 24 h, *SDHB* mRNA level was up regulated 33 fold in SKOV3 (*P* < 0.001) (Figure 4A). Under the same condition, SDHB protein expression was increased after 48 h transfection (Figure 4A). CCK8 was used to examine the cell viability. Over-expression of *SDHB* significantly inhibited ovarian cancer cell proliferation (Figure 4B). Likewise, Bcl-2 was decreased in *SDHB*-overexpressed SKOV3 cells (Figure 4C). These results revealed that overexpression of *SDHB* could inhibition cell growth in SKOV3 cancer cells.

### Overexpression of *SDHB* inhibited invasion and migration in SKOV3 cells

*SDHB* overexpression inhibited SKOV3 cell invasion (*P* < 0.05) and migration (*P* < 0.05) ability (Figure 4D). Down-regulation of p-FAK and MMP-2 expression was also found in *SDHB* overexpressed SKOV3 cells (Figure 4E).

### *SDHB* affected HIF-1α level in ovarian cancer cells

**Figure 5:**
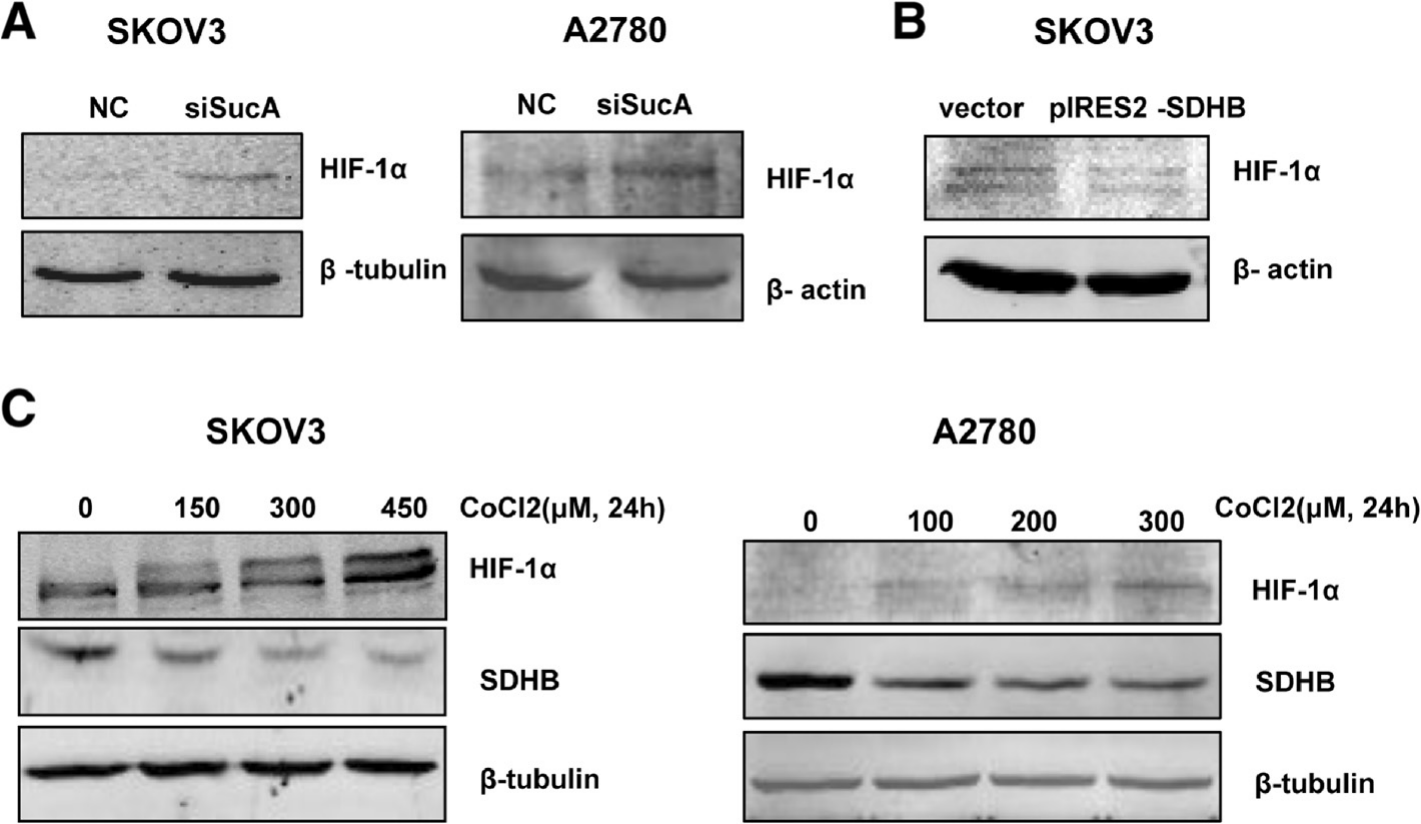
***SDHB*** **modulated HIF-1α expression in ovarian cancer cells.** SKOV3 and/ or A2780 cells were treated with *SDHB* siRNA **(A)** or over-expression plasmid **(B)** for 48 h, HIF-1α expression was analyzed by western blot with β-actin or β-tubulin as a loading control. **(C)** SKOV3 and A2780 cells were treated with CoCl2 at indicated concentrations for 24 h, HIF-1α and SDHB were examined by western blot.

Mitochondrial dysfunction repressed the protein synthesis of HIF-1α as well as reduced its trans-activation activity through AMPK signalling in human hepatoma HepG2 cells [37]. HIF-1α was up regulated in *SDHB*-silenced SKOV3 and A2780 cells, but down regulated in *SDHB*-overexpressed SKOV3 cells (Figure 5A,B). Consistent with these results, SDHB protein level was down regulated and conversely correlated with HIF-1α in hypoxic conditions induced by CoCl2 in ovarian cancer cells in a dose dependent manner (Figure 5C). To verify the effect of *SDHB* on energy metabolism in ovarian cancer cells, p-AMPKα was also analysed in *SDHB* over expressed SKOV3 cells. The level of p-AMPKα was decreased compared to the control (Figure 4F). These results showed SDHB might affect ovarian cancer cell phenotype *via* AMPK-HIF-1α signalling pathway.

### SDHB expression in human ovarian carcinoma and normal ovarian epithelium tissues

**Figure 6:**
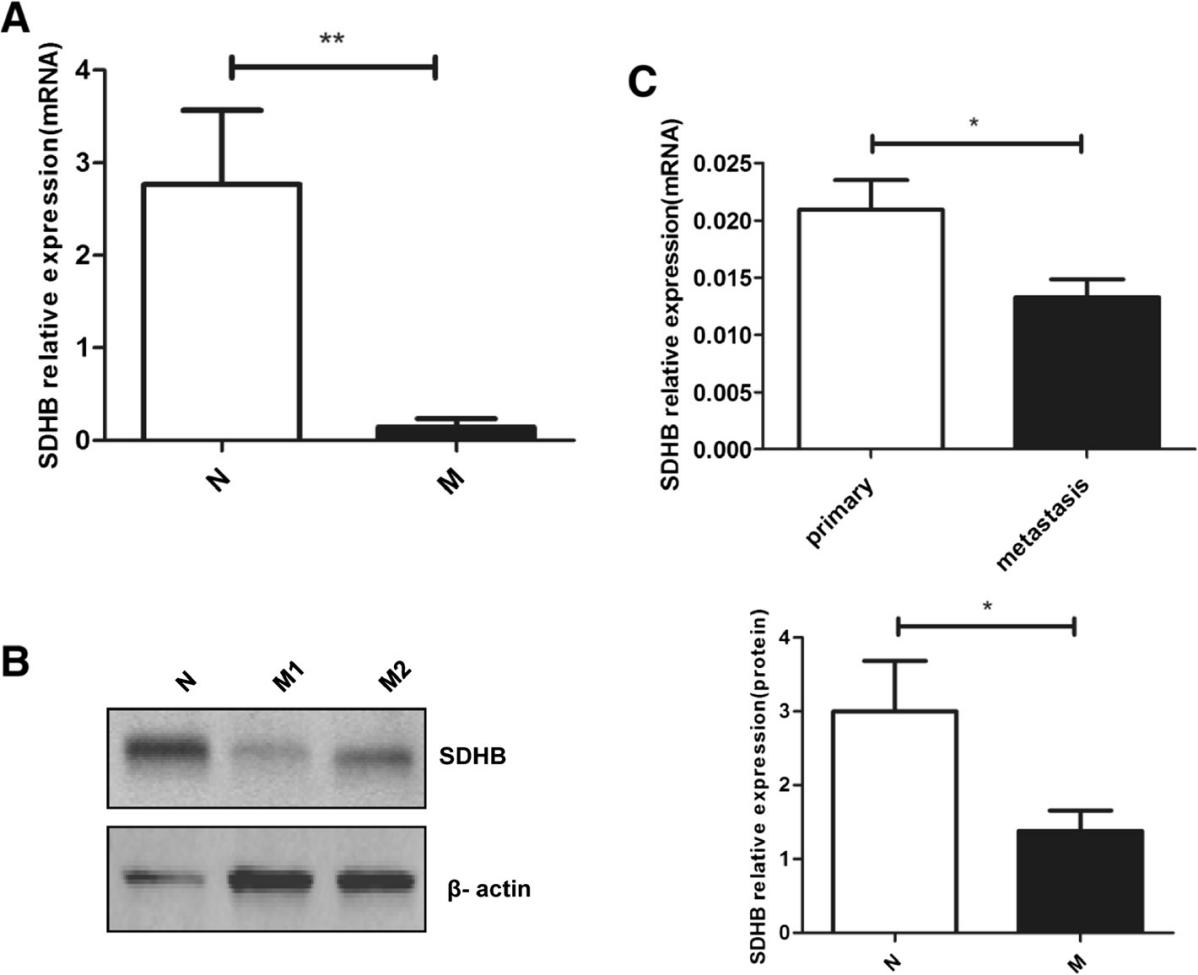
**Different expression of SDHB in human ovarian carcinoma tissues. (A)** The mRNA expression of *SDHB* in ovarian carcinoma tissues (n = 18) and normal ovarian epithelium tissues (n = 7) by real-time PCR (N = normal ovarian epithelium, M = ovarian carcinoma). **(B)** SDHB protein level in ovarian carcinoma and normal ovarian epithelium by western blot, M1 and M2 represent different patients. **(C)** *SDHB* mRNA expression in primary ovarian carcinoma (n = 7) and the corresponding metastasis (n = 7) by real-time PCR. Mean ± SEM. *P < 0.05 and **P < 0.01.

To confirm SDHB expression in normal and malignant ovarian tissues, mRNA and protein level of SDHB were determined. *SDHB* mRNA and protein level was significantly down regulated in human ovarian carcinoma compared with the controls (Figure 6A,B). Interestingly, *SDHB* mRNA expression was 36.80% lower in the corresponding metastasis (*P* < 0.05) than that in the primary ovarian carcinoma tissues (Figure 6C). The level of SDHB in tissue specimens was consistent with our results shown above.

## Discussion

In our study, we aimed to gain a better understanding of the role of SDHB in ovarian carcinoma by gene silencing and over expression. SDHB is one of the subunits of SDH takes parts in TCA [5], ATP is an indicator of energy metabolism. We found that the level ATP was decreased in *SDHB*-silenced cancer cells. On the other hand, p-AMPKα and p-P38 MAPK level were increased in the *SDHB*-silenced cells, suggesting that *SDHB* silencing could activate AMPK pathway in ovarian cancer.

SDHB protein is significantly lower compared with other SDH subunits in colorectal cancer tissues [10]. Notably, reduced SDHB expression in tumour tissues is associated with tumour de-differentiation. Restoration of SDHB inhibits the growth of cancer cells [10]. In our current study, *SDHB*-silenced cells increased proliferation in SKOV3 and A2780 cells, accompanied with elevated p-ERK level. Bcl-2 is functioned to oppose the apoptosis pathway of programmed cell death [38,39]. Effector caspases are responsible for initiating the events that lead to the hallmarks of apoptosis. Caspase-9, an essential initiator caspase required for apoptosis through mitochondrial pathway, is activated on the apoptosome complex, which directly resulted in cleave and activate effector caspases, such as caspase-3 [40,41]. Anti-apoptotic Bcl-2 level was increased and cleaved caspase 3 was decreased after treated with or without DDP in *SDHB*-silenced SKOV3 and A2780 cells.

FAK and MMPs are associated with metastases [42], secretion and activation of MMP-2 may be responsible for increased motility, invasiveness and metastasis of malignant cells [43]. In addition, increased FAK expression and activity frequently correlate with metastatic disease and poor prognosis [44]. We found that *SDHB* silencing promoted ovarian cancer invasion and migration accompanied with up-regulated expression of MMP-2 and p-FAK. To explore the role of SDHB in human ovarian carcinoma, we designed plasmid to induce SDHB expression in SKOV3 cells. *SDHB* overexpression inhibited cell proliferation in SKOV3. Cellular invasion and migration were inhibited in *SDHB*-over-expressed cells accompanied with reduced expression of p-FAK and MMP-2. These observations further verified the role of SDHB in ovarian carcinoma.

The relationship between HIF-1α and SDHB is controversial in tumourigenesis. SDHB is one of the subunits of SDH which takes part in TCA cycle and respiratory chain [5], AMPK could modulate metabolic stresses such as hypoxia. Study showed mitochondrial dysfunctions could result in reduced HIF-1α protein synthesis through AMPK-dependent manner in HepG2 cells [37]. Hypoxia is a characteristic of many malignancies arising from various sites [45] and HIF-1α is a hypoxia responsive factor [16]. Research reported that HIF-1α was up-regulated in chronically *SDHB*-silenced Hep3B cells [24], HIF-1α was overexpressed in *SDH*-deficient leiomyomas and renal cell cancer (HLRCC) [15]. While there was also report that increased HIF-1α was not associated with loss of SDHB expression on a series of familial and sporadic tumours [11]. In order to find out the key factor contributed to the phenomenon in our experiment, HIF-1α was analysed in treated cancer cells. The expression of HIF-1α was strongly elevated in *SDHB*-silenced ovarian cancer cells. Moreover, SDHB was decreased in CoCl2-treated cancer cells accompanied by HIF-1α up-regulation. In accordance with these results, over-expressed *SDHB* could inhibit HIF-1α expression. The results showed *SDHB* affected ovarian cancer progression by altering HIF-1α expression. It was suggested that *SDHB* silencing up-regulated HIF-1α *via* activation of AMPKα in cancers in accordance with the aerobic glycolysis (Warburg effect) [46]. To verify the effect of *SDHB* on energy metabolism in ovarian cancer, p-AMPKα was also analysed in *SDHB*-overexpressed SKOV3 cells. The level of p-AMPKα was decreased compared to control. In addition, HIF-1α is decreased by inhibiting ERK pathway in cervical carcinoma CaSki cells [47], but apoptosis is enhanced by HIF-1α knockdown in pancreatic cancerous BxPC-3 cells [48]. HIF-1α also promotes cell migration by regulating MMP-2 [49] and FAK [50], which is in line with our current data. These results showed SDHB might affect cancer cell proliferation, invasion, migration, and apoptosis *via* AMPK-HIF-1α in ovarian carcinoma.

Finally, we compared the expression of SDHB between ovarian carcinoma and normal ovarian epithelium tissues. SDHB mRNA and protein was decreased in human ovarian carcinoma compared with normal ovarian epithelium. In addition, SDHB mRNA expression was decreased in the corresponding metastasis compared with the primary ovarian carcinoma, suggesting SDHB contributed to tumour metastasis. Moreover, it was well known that SDHB-associated PCCs/PGLs often metastasise in a familial setting [11]. Our current data suggested that SDHB play an important role in cancer progression. Enhanced SDHB expression inhibited cell proliferation, invasion, migration and promoted apoptosis in human ovarian carcinoma. The relationship between SDHB expression and clinical characteristics in human ovarian carcinoma will be determined in our future studies.

## Conclusions

In current study, we demonstrated that SDHB played an important role in cellular proliferation, invasion, migration and apoptosis *via* AMPK-HIF-1α pathway in human ovarian carcinoma. Overexpression of SDHB might be an effective therapeutic strategy for treatment of ovarian carcinoma.

## Additional file

Additional file 1: Figure S1. SDHB expression in SKOV3 and A2780. (A, B) Real-time PCR and western blot were used to analyse *SDHB* mRNA and protein expression in SKOV3 and A2780 cells.

## Abbreviations

MMP-2: Matrix metalloproteinase-2
FAK: Focal adhesion kinase
Bcl-2: B-cell lymphoma-2
CoCl2: Cobalt chloride
DDP: Cis-platinum
RIPA: Radio immuno-precipitation assay
PMSF: Phenylmethanesulfonyl fluoride
TBST: Tris-buffered saline with Tween-20
NC: Nitrocellulose membrane
PVDF: Polyvinylidene fluoride
