# Supplementary material for: Succinate dehydrogenase subunit B inhibits the AMPK-HIF-1α pathway in human ovarian cancer in vitro
Source: J Ovarian Res. 2014 Dec 10;7:115. doi: 10.1186/s13048-014-0115-1 (PMC4279696; doi:10.1186/s13048-014-0115-1)
Supplement: Additional file 1: Figure S1. — SDHB expression in SKOV3 and A2780. (A, B) Real-time PCR and western blot were used to analyse SDHB mRNA and protein expression in SKOV3 and A2780 cells. [file 13048_2014_115_MOESM1_ESM.pdf]

Additional file 1

Figure S1.

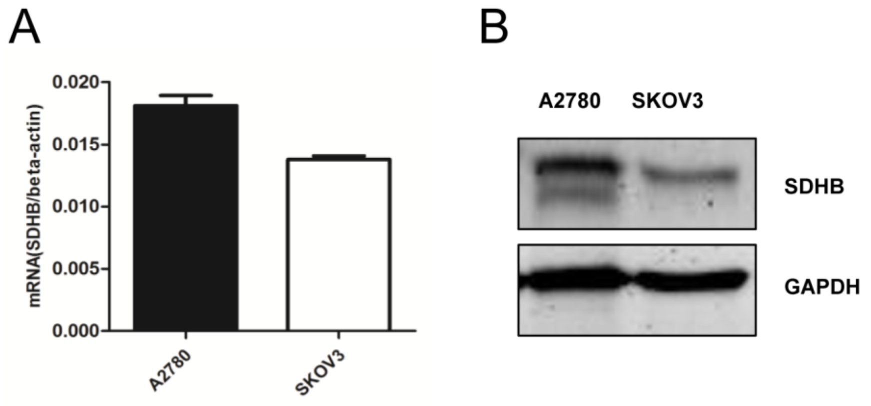

Figure S1. SDHB expression in SKOV3 and A2780. (A, B) Real-time PCR and western blot were used to analyse *SDHB* mRNA and protein expression in SKOV3 and A2780 cells.
